# TBHQ-Overview of Multiple Mechanisms against Oxidative Stress for Attenuating Methamphetamine-Induced Neurotoxicity

**DOI:** 10.1155/2020/8874304

**Published:** 2020-11-27

**Authors:** Yuan-Ling Zhao, Wei Zhao, Ming Liu, Lian Liu, Yun Wang

**Affiliations:** ^1^Department of Clinical Pharmacology, School of Pharmacy, China Medical University, Shenyang, Liaoning 110122, China; ^2^Department of Drug Control, Criminal Investigation Police University of China, Shenyang, Liaoning 110854, China

## Abstract

Methamphetamine is a derivative of amphetamines, a highly addictive central stimulant with multiple systemic toxicity including the brain, heart, liver, lung, and spleen. It has adverse effects such as apoptosis and breakdown of the blood-brain barrier. Methamphetamine is a fatal and toxic chemical substance, and its lethal mechanism has been widely studied in recent years. The possible mechanism is that methamphetamine can cause cardiotoxicity and neurotoxicity mainly by inducing oxidative stress so as to generate heat, eliminate people's hunger and thirst, and maintain a state of excitement so that people can continue to exercise. According to many research, there is no doubt that methamphetamine triggers neurotoxicity by inducing reactive oxygen species (ROS) production and redox imbalance. This review summarized the mechanisms of methamphetamine-induced neurotoxicity including apoptosis and blood-brain barrier breakdown through oxidative stress and analyzed several possible antioxidative mechanisms of tert-butylhydroquinone (TBHQ) which is a kind of food additive with antioxidative effects. As a nuclear factor E2-related factor 2 (Nrf2) agonist, TBHQ may inhibit neurotoxicity caused by oxidative stress through the following three mechanisms: the nicotinamide adenine dinucleotide phosphate (NADPH) oxidase system, the astrocytes activation, and the glutathione pathway. The mechanism about methamphetamine's toxic effects and its antioxidative therapeutic drugs would become a research hotspot in this field and has very important research significance.

## 1. Introduction

According to data from 2018, the global seizures of methamphetamine reached 228 tons and increased by 23% compared with the previous years. Methamphetamine abuse has been in the first place among amphetamine-type stimulants. During the period 2014-2018, global seizures of methamphetamine accounted for 71% of the total amphetamine-type stimulants, followed by amphetamine with 21% [1]. Therefore, related studies on the prevention and treatment of methamphetamine addiction have become increasingly important. Brain hyperthermia is an important death cause of methamphetamine. Toxic doses of methamphetamine can cause hyperthermia, and high core temperature can cause a large amount of dopamine loss in the brain. Experiments showed that hyperthermia was one of the causes of methamphetamine neurotoxicity [2–4]. Methamphetamine induces dose-dependent brain hyperthermia. Compared with the core of the body, the brain warms up faster and to a higher degree [5, 6]. At an ambient temperature of 23°C, 9 mg/kg methamphetamine made brain temperature (nucleus accumbens) and muscle temperature (temporal muscle) increased to 38.92 ± 0.34°C and 37.92 ± 0.32°C, respectively [7]. Brain hyperthermia may play a role in destroying neural cells, causing brain edema, increasing the permeability of the blood-brain barrier, and eventually lead to apoptosis and the blood-brain barrier breakdown [8]. Fish were exposed to a suitable temperature (25 ± 1°C) and a high temperature (32 ± 1°C). The study found that under high temperature, the concentration of antisuperoxide anion free radicals and the activity of superoxide dismutase (SOD) were significantly reduced, proving that hyperthermia caused oxidative stress [9]. Another study found that the ROS and reactive nitrogen (RNS) levels in oysters exposed to a high temperature (32°C) were significantly increased, and the incidence of apoptosis was significantly increased [10]. The concrete results were that the contents of protein carbonyl (an indicator of ROS) were increased 11.7 times, the intensity of nitrotyrosine protein (an indicator of RNS) was increased 3.5 times, and the activity of the apoptosis indicator caspase-3/7 was increased 5 times [10]. A scholar randomly selected 96 methamphetamine abusers to measure the plasma iron-reducing ability and the serum malondialdehyde (MDA) content to assess the total antioxidant capacity and lipid peroxidation status in the body [11]. The statistical analysis showed that the total antioxidant capacity in the methamphetamine group (0.31 ± 0.04 *μ*mol/L) was significantly lower than the control group (0.46 ± 0.05 *μ*mol/L), and the level of MDA in the methamphetamine group (4.38 ± 5.05 *μ*mol/L) was significantly higher than the control group (1.72 ± 2.04 *μ*mol/L) [11]. The scholar's research showed that abuse of methamphetamine enhanced the level of oxidative stress and lipid peroxidation in the body. The neurotoxicity of methamphetamine is mainly produced by inducing oxidative stress, so antioxidation is of great significance in the prevention and treatment of methamphetamine toxicity. This review briefly explains the neurotoxicity of methamphetamine from apoptosis and blood-brain barrier breakdown, and summarizes the possible antioxidative mechanism of TBHQ from three perspectives. At present, there are only a few related literatures about the effects of TBHQ on methamphetamine, and this review is expected to open up new ideas for the prevention and treatment of methamphetamine neurotoxicity.

## 2. Methamphetamine and Neurotoxicity

Methamphetamine can induce hyperthermia, thereby increasing the level of oxidative stress at the dopamine terminal and ultimately causing neurotoxicity [12–14]. The hyperthermic reaction induced by methamphetamine can affect the oxidation process of dopamine from the following two aspects: (1) In the autooxidation process of dopamine, free iron and other transition metals play an inducing role [15]. Methamphetamine can promote the release of free iron by inducing a hyperthermic reaction [16] and ultimately increase the autooxidation level of dopamine. (2) During the enzymatic oxidative degradation of dopamine, hyperthermia may cause enzyme activation [12], thereby accelerating the enzyme reaction process and accelerating the degradation of dopamine. The oxidation products of dopamine include dopamine quinone and free radicals [12]. The production of free radicals is a key factor in inducing methamphetamine neurotoxicity. A large amount of ROS causes oxidative stress damage to various cells in the central nervous system so as to trigger neurotoxicity [17–19]. Excessive nitric oxide (NO) causes neurotoxicity by affecting the normal energy supply of mitochondria [20]. The formation of NO and superoxide (O_2_^−^) and the peroxynitrite (ONOO^−^) synthesized by both trigger long-term neurotoxicity by affecting the outflow of glutamate, increasing the concentration of extracellular glutamate, and excessively stimulating glutamate receptors [21]. The neurotoxicity induced by methamphetamine can be reflected in two aspects: apoptosis and blood-brain barrier breakdown.

### 2.1. Methamphetamine and Apoptosis

Methamphetamine causes damage to mitochondria in the brain, thereby leading to apoptosis and a series of other neurotoxicities. Oxidative stress is an important link between mitochondrial damage and methamphetamine-induced neurotoxicity. Studies showed that methamphetamine interfered with mitochondrial energy metabolism by inhibiting the Krebs cycle and electron transport chain to ultimately cause neurotoxicity [22–24]. After methamphetamine administration, the activity of electron transport chain complex IV (cytochrome oxidase) decreased [25]. Other studies showed that a large amount of methamphetamine in a short time significantly reduced the activity of electron transport chain complex II (succinate dehydrogenase) [26–28]. The mechanism of inhibiting the electron transport chain complex is mainly due to the large amount of ROS and RNS produced by intensive oxidative stress after methamphetamine exposure. ROS and RNS directly act on the electron transport chain complex, reducing their activity. At the same time, the inhibition on the electron transport chain complex increases the leakage of electrons and produces O_2_^−^ to form a positive feedback loop [29]. The damaged mitochondria are subsequently degraded by autophagy [30]. Many studies showed that methamphetamine-induced mitochondrial damage enhanced susceptibility to proapoptosis [22, 31–33]. Methamphetamine exposure causes intensive oxidative stress, and the produced ROS and RNS can block the electron transport chain of mitochondria so that the energy metabolism of mitochondria is interfered to cause the damage of mitochondria. Mitochondria damage induces neurotoxicity such as apoptosis. Therefore, oxidative stress and ROS are important factors for methamphetamine-induced apoptosis. In addition, PUMA, PKC*δ*, miRNA, and lncRNA also seem to be involved in the process of methamphetamine-induced neurocyte apoptosis [29].

### 2.2. Methamphetamine and Blood-Brain Barrier Breakdown

The blood-brain barrier is a barrier between plasma and brain cells, and its role is to prevent certain substances from entering the brain tissue from the blood. The innermost layer of the blood-brain barrier is vascular endothelial cells. The two endothelial cells rely on tight junction proteins to connect and closely overlap each other. There are some pericytes outside the endothelial cells. The outer side of the endothelial cells and pericytes is covered by a basement membrane. The outermost layer of the blood-brain barrier consists of astrocytic end-feet. Among these structures, closely overlapping endothelial cells are the most important structure of the blood-brain barrier. Tight junction proteins include claudin-5, ZO-1, and occludin. Methamphetamine disrupts the tight overlap of the endothelium by downregulating or redistributing these tight junction proteins, thereby increasing endothelial permeability and making the blood-brain barrier collapse. Endothelial cells are very sensitive to the redox imbalance induced by methamphetamine, and endothelial cells produce ROS through oxidative stress [34]. There are many reasons for methamphetamine to cause oxidative stress. Methamphetamine downregulates glutamate-cysteine ligase (GCL) [35]. GCL is the rate-limiting enzyme for the synthesis of the antioxidant glutathione [36]. Methamphetamine can also affect the outflow of glutamate, increasing the concentration of extracellular glutamate [21]. Glutamate is one of the raw materials of glutathione, and it combines glycine and cysteine to form glutathione [37]. The outflow of glutamate reduces its intracellular concentration, thereby reducing the content of glutathione. Methamphetamine reduces the concentration of glutathione through the above two factors, which causes redox imbalance and ROS production. Methamphetamine can also induce oxidative stress through other two pathways, activating NADPH oxidase or making astrocytes overactivated. Oxidative stress and ROS can cause intracellular protein thiol oxidation [38], which can activate nonmuscle myosin light chain kinase (nmMLCK) [39]. The activation of nmMLCK causes the phosphorylation of claudin-5 and occludin, makes them lose their functions, weakens the connection between endothelial cells, and reduces the barrier function [40]. After methamphetamine administration, the blood-brain barrier is eventually destroyed by the series of effects mentioned above.

## 3. TBHQ and Antioxidative Stress

TBHQ is a food antioxidant. Many research showed that TBHQ reduced the level of oxidative stress in mammals [35, 41–43]. Antioxidant response elements are mainly mediated by Nrf2 and can participate in the transcriptional regulation process of phase II detoxification enzymes and antioxidant proteins, including quinone oxidoreductase, glutathione S-transferase (GST), and GCL. [44]. Nrf2 is one of the key factors to prevent excessive oxidative stress in brain cells. TBHQ, as an Nrf2 agonist [45], mainly plays a role in inhibiting oxidative stress in the brain through the following mechanisms: On one hand, Nrf2 is responsible for activating transcription in response to oxidative stress. In the presence of a large number of stimuli, Nrf2 moves from the cytoplasm to the nucleus, and in turn combines with antioxidant response elements [46], so TBHQ can play an antioxidative stress role by activating Nrf2. On the other hand, antioxidant response elements/electrophile response elements are activated transcription by TBHQ, and the increased gene expression caused by this process can also prevent excessive oxidative stress [47]. Research data showed that TBHQ increased the levels of glutathione and GCL in astrocytes and neurons, while in astrocytes, the increase in both was greater [48]. In astrocytes, after treatment with 20 *μ*M TBHQ, glutathione levels were increased by 50% and GCL activity increased by 150%. However, in neurons, when treated with 20 *μ*M TBHQ, glutathione levels only increased by 20%, and GCL activity only increased by 40% [48]. This phenomenon may be due to TBHQ activating Nrf2, and the Nrf2 pathway has been shown to regulate glutathione metabolism [49]. In addition, another study showed that TBHQ moderately increased the number of astrocytes in the brain, and in the central nervous system, astrocytes played an extremely important role in antioxidative stress [50]. NADPH, also known as quinone oxidoreductase 1 (NQO1), can accelerate quinone excretion by reducing quinone to hydroquinone; however, in the absence of this enzyme, quinone is reduced to hemihydroquinone, which generates ROS through redox. NADPH oxidase system is one of the sources of ROS [34]. Nrf2 may resist oxidative stress by downregulating or inhibiting the expression of NADPH oxidase. Therefore, TBHQ plays an antioxidative role by activating Nrf2.

### 3.1. TBHQ against Oxidative Stress through NADPH Oxidase System

**Figure 1:**
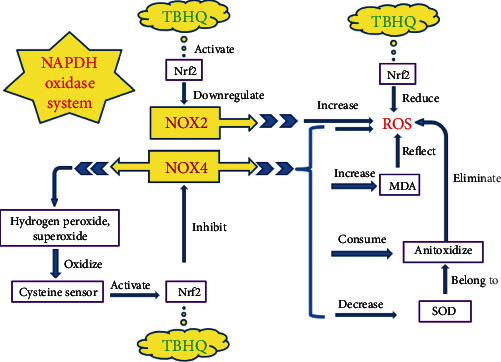
NADPH oxidase system and antioxidative stress. NADPH oxidase system contains NOX2 and NOX4. TBHQ activates Nrf2, then Nrf2 negatively regulates NOX2, and NOX2 can produce ROS, and Nrf2 can also directly reduce ROS. Under homeostasis, NOX4 produces hydrogen peroxide and superoxide which both oxidize cysteine sensor and cysteine sensor activate Nrf2; then, Nrf2 inhibits the transcription of NOX4, making NOX4 under homeostasis, forming a positive feedback loop. However, with upregulation of NOX4, the homeostasis and feedback loop mentioned above are destroyed, creating a prooxidative environment; then, ROS and MDA will increase, and MDA can indirectly reflect ROS. In addition, NOX4 consumes and inhibits antioxidases including SOD. NADPH: nicotinamide adenine dinucleotide phosphate; TBHQ: tert-butyl hydroquinone; Nrf2: nuclear factor E2 related factor 2; ROS: reactive oxygen species; SOD: superoxide dismutase; MDA: malondialdehyde; NOX: NADPH oxidase.

TBHQ exerts an antioxidative stress effect through the NADPH oxidase system, and the mechanism is shown in Figure 1. NADPH oxidase family includes 7 isoforms of NOX1, NOX2, NOX3, NOX4, NOX5, DUOX1, and DUOX2. NADPH oxidase is a membrane protein, mainly responsible for the transmission of electrons, transferring electrons to molecular oxygen, generating ROS and O_2_^−^[51]. All NADPH oxidase isoforms act as catalysts, transferring two electrons from NADPH to molecular oxygen through its FAD domain and two heme repair groups [51, 52]. NOX1, NOX2, NOX3, and NOX5 produce O_2_^−^, and NOX4, DUOX1, and DUOX2 release hydrogen peroxide [51, 53, 54]. NADPH oxidase can be stimulated and activated by many factors and subsequently produces a large amount of ROS, including drug factors (e.g., methamphetamine), hormones, and environmental factors (e.g., noise stimulation). NOX2 is highly expressed in the cells of the central nervous system, including cerebrovascular endothelial cells. Endothelial NOX2 has low activity before being activated (i.e., under physiological conditions), and overexpressed NOX2 after activation can cause endothelial cells to produce a large amount of ROS, resulting in the occurrence of oxidative stress. It was found that ROS derived from NOX2 caused severe oxidative damage to neurocytes and cerebrovascular endothelium [55]. NOX2 deficiency prevents brain oxidative stress, reverses the production of cerebrovascular O_2_^−^, and reverses the functional damage of endothelial cells, making it tend to normalize [56]. NOX4 also plays a very important role in the regulation of oxidative stress [57]. The increased expression of NOX4 in the brain leads to increased production of ROS, which not only causes excessive consumption of endogenous antioxidase in the brain but also reduces its activity, thereby weakening the brain's ability to scavenge ROS. Excessive ROS may also cause oxidation of proteins and fats, cause damage to DNA, and affect energy metabolism, thereby eventually inducing neuronal death and apoptosis in the brain [58]. SOD scavenges oxygen free radicals and blocks the pathological chain reaction. It is an important defending enzyme, and its activity has become a key index for measuring the scavenging ability of oxygen free radicals [58, 59]. Oxygen free radicals attack cells and produce large amounts of lipid peroxides, including MDA, and MDA can be used as an important index to indirectly reflect changes in free radical content [58, 60]. A study found that upregulating NOX4 increased oxygen free radicals, decreased the SOD expression, and increased the MDA expression [58].

NADPH oxidase is present in cells capable of producing ROS (including neurons, glial cells, macrophages) in the brain and is considered to be an important factor for the generation of ROS and the maintenance of ROS homeostasis [61]. The two isoforms NOX2 and NOX4 of the NADPH oxidase system are involved in the process of brain oxidative damage. The knockout of Nrf2 upregulates ROS of mitochondrial origin, and this process involves oxidative phosphorylation. Nrf2 can also prevent the oxidation of mitochondrial fatty acids, and at the same time regulate the availability of substrates, thereby affecting the cellular bioenergetics [62, 63]. This process involves oxidative phosphorylation and ROS production. Nrf2 not only interferes with the production of ROS in the mitochondria but also affects the process of ROS production by NADPH oxidase. Experiments showed that the specific regulation mechanism of Nrf2 was related to NOX2 and NOX4 of the NADPH oxidase system [64–66]. The mechanism of Nrf2 acting on NADPH oxidase is complex, and it may be through direct and indirect regulation through promoter binding and chromatin remodeling [64]. The expression of NOX2 was upregulated in primary brain hippocampal glio-neuronal cultures of Nrf2-KO mice, indicating that Nrf2 negatively regulated NOX2 [64]. A positive feedback loop is formed between Nrf2 and homeostatic NOX4. Activated Nrf2 inhibits the NOX4 transcription [65], and then, homeostatic NOX4 produces hydrogen peroxide and O_2_^−^, which subsequently oxidize the cysteine sensor, and the cysteine sensor can activate Nrf2 [66] to form a positive feedback loop. However, in the absence of Nrf2, NOX4 homeostasis is disrupted, and there is no such feedback loop. Overexpressed NOX4 consumes antioxidase and increases ROS production [64]. Therefore, TBHQ, as an Nrf2 agonist, plays a role in reducing ROS and resisting oxidative stress.

### 3.2. TBHQ against Oxidative Stress by Regulating Astrocytes

**Figure 2:**
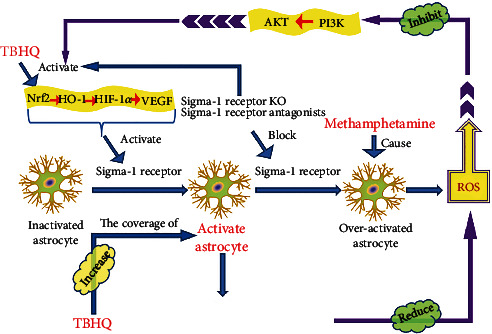
Double-edged sword effects of astrocyte activation. TBHQ activates Nrf2 and then regulates its downstream HO-1, HIF-1*α*, and VEGF. This process activates the Sigma-1 receptor, so that astrocytes become activated from inactivated, and activated astrocytes can play a role in resisting methamphetamine-induced oxidative stress. TBHQ can also increase activated astrocytes and their coverage to reduce ROS. ROS inhibits the PI3K/AKT signaling pathway. TBHQ, as an Nrf2 agonist, can also indirectly activate Nrf2 by positively regulating the PI3K/AKT signaling pathway. Sigma-1 receptor KO or Sigma-1 receptor antagonists can activate Nrf2 as well as block excessive Sigma-1 receptors, thereby preventing the overactivation of astrocytes. However, methamphetamine can induce overactivation of astrocytes. TBHQ: tert-butyl hydroquinone; Nrf2: nuclear factor E2 related factor 2; HO-1: heme oxygenase-1; HIF-1*α*: hypoxia-inducible factor-1*α*; VEGF: vascular endothelial growth factor; KO: knockout; ROS: reactive oxygen species; PI3K: phosphatidylinositol 3-kinase; AKT: protein kinase B.

TBHQ plays an antioxidative stress role by regulating astrocytes, and the mechanism is shown in Figure 2. Astrocytes are an important part of the blood-brain barrier. They secrete a variety of neuroactive molecules and respond to a variety of immune regulatory signals to counteract methamphetamine-induced oxidative stress [67]. The outermost layer of the blood-brain barrier is covered by astrocytic end-feet. A study found that in the blood-brain barrier coculture model, the removal of astrocytes destroyed the tight junctions of the blood-brain barrier and led to increased permeability [68]. Astrocytes express glutamatergic, GABAergic, adrenergic, purinergic, serotonergic, muscarinic, and peptidergic receptors [67, 69]. Activated astrocytes release glutamate, prostaglandins, ATP and NO, and other neuroactive molecules [67, 70]. Changes in astrocyte activity directly affect the central nervous system [71], and the regulatory mechanism of astrocytes is complex. Abnormal activity or function of astrocytes can promote nervous system damage. Sigma-1 receptor is closely related to methamphetamine-induced neurotoxicity. Recent studies have found that Sigma-1 receptor antagonists can reduce methamphetamine-induced oxidative stress, cerebral hyperthermia, and behavioral abnormalities [67, 72, 73]. Glial fibrillary acidic protein (GFAP) is an index of astrocyte activation. After knocking out the Sigma-1 receptor, it was found that GFAP expression was abrogated [74]. Both Sigma-1 receptor antagonists BD1047 and SN79 can block the overactivation of astrocytes and attenuate the expression of proinflammatory cytokines after methamphetamine exposure [74–76]. Methamphetamine can regulate the Sigma-1 receptor in astrocytes so as to change astrocyte activity [76]. A study showed that methamphetamine simultaneously increased the oxidative burden and antioxidative capacity of astrocytes. This effect may be exerted through abnormal regulation of mitochondria. Under physiological conditions, astrocytes protect the central nervous system from damage by maintaining redox homeostasis and a delicate balance. Stimulation of methamphetamine can lead to dichotomous dysregulation of redox. Attenuating methamphetamine-mediated secondary messenger signaling downstream of the Sigma-1 receptor can target dysregulation of mitochondrial regulatory proteins in astrocytes [77]. Heme oxygenase-1 (HO-1) is a phase II antioxidative enzyme, and its expression level can be regulated by TBHQ and Nrf2. Another study showed that inducing the activation of Nrf2 and the expression of HO-1 in astrocytes downregulated the expression of proinflammatory cytokines and upregulated the antioxidative mechanism [78]. Phosphatidylinositol 3-kinase (PI3K) is also involved in resisting methamphetamine-induced oxidative stress. PI3K works by activating protein kinase B (also known as AKT) [79]. PI3K/AKT has been reported as the upstream signaling pathway of Nrf2 in many papers [79–81].

A recent experiment showed that in astrocytes, the Sigma-1 receptor was activated by the Nrf2/HO-1 signaling pathway, thereby making astrocytes from inactivation to activation and then reducing the production of ROS [82]. The activation of Nrf2 and HO-1 partially mediates the activation of Sigma-1 receptor and its anti-inflammatory and antioxidative effects [82]. The Sigma-1 receptor is equivalent to a molecular chaperone and participates in various mental diseases by interacting with multiple protein or lipid molecules. Knockout or loss of Sigma-1 receptors may activate the Nrf2/HO-1 signaling pathway. Astrocytes and neurons contain a large number of Sigma-1 receptors, and their silencing will induce a decrease in mitochondrial membrane potential and aberrant formation of mitochondrial aggregates [83–85]. Astrocyte activation and overactivation show double-edged sword effects in multiple mental diseases [86]. After methamphetamine administration, Sigma-1 receptor activates astrocytes through a self-activation mechanism [76]. Methamphetamine exposure makes astrocytes overactivated. Sigma-1 receptor exerts neuroprotective effects by maintaining homeostasis of astrocytes and neurons and maintaining the proper and balanced degree of activation of astrocytes. Knock-out of Sigma-1 receptor leads to the imbalance of astrocyte populations and enhancement of the Nrf2 signaling pathway, which can attenuate excessive oxidative stress, promote neuronal survival, and reduce methamphetamine-induced neurotoxicity [83].

PI3K has both Ser/Thr kinase activity and phosphatidylinositol kinase activity [87]. The Ser/Thr kinase activity of PI3K can activate its downstream target AKT [79]. PI3K/AKT signaling pathway can phosphorylate Ser/Thr residues, which is a key for Nrf2 activation [79, 81]. Under hyperoxia conditions, PI3K is inhibited. In the presence of a large amount of ROS, Nrf2 and its downstream are inhibited through the PI3K/AKT signaling pathway [79]. Methamphetamine leads to the overactivation of astrocytes and generates ROS, thereby inhibiting the expression of Nrf2. This process can be reversed by TBHQ. TBHQ reverses methamphetamine-induced oxidative stress damage by directly activating the Nrf2/HO-1 signaling pathway and indirectly regulating Nrf2 through the PI3K-AKT signaling pathway [79]. Hypoxia-inducible factor-1*α* (HIF-1*α*) and vascular endothelial growth factor (VEGF) are downstream of the Nrf2/HO-1 signaling pathway. An experimental result showed that TBHQ activated astrocytes and increased astrocytic end-feet coverage by activating the Nrf2/HO-1/VEGF pathway [88]. The antioxidative stress and neuroprotective effect of astrocytes are closely related to the degree of activation. Both inactivated and overactivated astrocytes can induce dysfunction. TBHQ increases the nuclear accumulation of Nrf2 and at the same time enhances the expression of antioxidative genes downstream of Nrf2 (including HO-1 and NQO1), moderately activates astrocytes, and reduces the production of inflammatory cytokines, thereby reducing apoptosis and neuronal death [89]. Under physiological conditions, Nrf2 binds to Keap1 to form the Nrf2 Keap1 complex [90]. Under oxidative stress, Nrf2 is released from Keap1's antioxidant response element; in this way, Nrf2 is activated. Meanwhile, Nrf2 achieves its accumulation in the nucleus by transferring from the cytoplasm to the nucleus, thereby playing a series of neuroprotective effects such as antioxidative stress [90, 91]. HO-1 is induced after Nrf2 activation and is a target gene for antioxidative stress [92, 93]. The Nrf2/HO-1 signaling pathway is closely related to oxidative stress in the brain [94–96] and some antioxidants work by upregulating Nrf2 and HO-1 [97–99]. The HIF-1*α*/VEGF pathway may be related to oxidative stress in astrocytes. The Nrf2/HO-1 signaling pathway may inhibit oxidative stress in astrocytes by regulating HIF-1*α* and VEGF [90]. HO-1 is a phase II antioxidative enzyme, and its expression level can be regulated by TBHQ. TBHQ promotes the translocation of Nrf2 to the nucleus, and Nrf2 participates in the regulation of HO-1 expression. PI3K/AKT signaling pathway is the upstream of Nrf2, and the protective effect of TBHQ on methamphetamine-induced neurotoxicity is also closely related to the activation of PI3K/AKT [79]. TBHQ regulates Sigma-1 receptors through the Nrf2/HO-1 and PI3K/AKT signaling pathways, thereby ultimately regulating the activation state of astrocytes and playing a role in resisting oxidative stress. In astrocytes, TBHQ increases the mRNA and protein levels of HO-1 by inducing a coordinated interaction between Nrf2 and c-Jun [100].

### 3.3. TBHQ against Oxidative Stress via Glutathione Pathway

**Figure 3:**
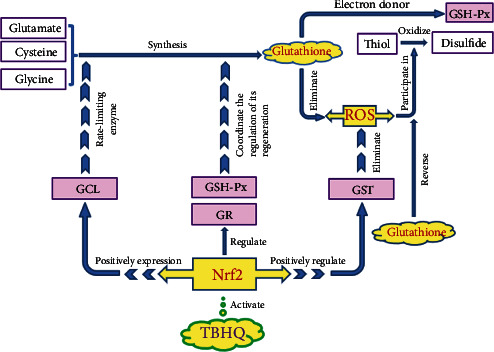
Glutathione pathway and antioxidative stress. Glutathione is synthesized by three substances glutamate, cysteine, and glycine. Glutathione can eliminate ROS and act as an electron donor for GSH-Px to reduce peroxides. TBHQ activates Nrf2, and Nrf2 positively regulates GST. GST is a ROS detoxification enzyme that eliminates ROS. At the same time, Nrf2 promotes the expression of GCL. GCL is the rate-limiting enzyme for glutathione synthesis. Nrf2 can also regulate GSH-Px and GR, which coordinate the regulation of glutathione regeneration. ROS is also involved in the oxidation of thiols to disulfides, which can be reversed by glutathione. TBHQ: tert-butyl hydroquinone; Nrf2: nuclear factor E2 related factor 2; GCL: glutamate-cysteine ligase; GSH-Px: glutathione peroxidase; GR: glutathione reductase; GST: glutathione S-transferase.

TBHQ can also inhibit the oxidative stress of cerebrovascular endothelial cells and neurocytes via the glutathione pathway, and the mechanism is shown in Figure 3. Glutathione is an important antioxidant in the body. It is a ROS scavenger, especially to clear the main form of ROS -O_2_^−^[101]. Glutathione is also the electron donor of glutathione peroxidase (GSH-Px) to reduce peroxides [102]. First, Nrf2 increases the expression of GCL [103], which is the rate-limiting enzyme for glutathione synthesis [36], so Nrf2 increases glutathione synthesis. Second, Nrf2 can also regulate GSH-Px and glutathione reductase (GR), and the two enzymes coordinate the regulation of glutathione regeneration. Third, Nrf2 can also positively regulate GST (ROS detoxification enzyme). Glutathione reduces ROS through antioxidative stress to resist neurocytes apoptosis and inhibit blood-brain barrier breakdown. In addition, glutathione can also reverse the process of thiol oxidation to disulfide in the presence of ROS (reversed by the reduction of disulfide through sulfhydryl/disulfide exchange) [38], thereby blocking the activation process of nmMLCK, blocking the middle link of blood-brain barrier breakdown, thereby protecting the blood-brain barrier from being destroyed. Therefore, the regulation of glutathione-related enzymes by Nrf2 has extremely important therapeutic significance for methamphetamine-induced oxidative stress. TBHQ regulates the above process by activating Nrf2, thereby reducing methamphetamine-induced neurotoxicity.

## 4. Concluding Remarks

Methamphetamine addiction and chronic poisoning have been widely concerned by society. The toxicity of methamphetamine mainly includes cardiotoxicity and neurotoxicity, and its toxicity is closely related to oxidative stress. There are also scholars who explored the lung toxicity of methamphetamine, and they found that its lung toxicity was also related to oxidative stress [104–106]. TBHQ is a food additive often used as an antioxidant or preservative. Many studies have confirmed that TBHQ can activate Nrf2 [43, 45, 46] and can play the role of antioxidative stress through Nrf2, but the specific mechanism is not very clear. This review synthesizes many scholars' research and systematically explains how TBHQ can resist methamphetamine-induced oxidative stress from three perspectives, including regulating the NADPH oxidase system, adjusting astrocytes activation, and regulating the glutathione pathway. Methamphetamine induces neurotoxicity including apoptosis and blood-brain barrier breakdown through oxidative stress. This review also briefly introduces the mechanisms of methamphetamine-induced neurotoxicity. Brain hyperthermia is a major cause of death from methamphetamine, and hyperthermia is one of the factors that produce excessive ROS and induce oxidative stress. All in all, the redox imbalance induced by methamphetamine is an important reason for its neurotoxicity, so the prevention and treatment of methamphetamine-induced neurotoxicity can be started from the aspect of antioxidation. As a food additive, TBHQ has almost no toxic and side effects. If the antioxidative effect of TBHQ can be used for the prevention and treatment of methamphetamine toxicity, it will provide a new and safer treatment for methamphetamine addiction. This review comprehensively and systematically introduces TBHQ, which is expected to provide new clues and develop new methods for the detoxification treatment of methamphetamine. As the number of methamphetamine users increases year by year, research and reviews in this area have increasingly important clinical significance.
